# Supplementary material for: Knowledge and stigma of autism spectrum disorders in Chinese university students in the context of inclusive education
Source: PLoS One. 2025 Jun 26;20(6):e0320033. doi: 10.1371/journal.pone.0320033 (PMC12200771; doi:10.1371/journal.pone.0320033)
Supplement: S1 File — (ZIP) [file pone.0320033.s001.zip › figure.doc]

**Table 1. Demographic statistics of participants (*N* = 2,081)**

| **Characteristics** |  | ***N*（%）** |
| --- | --- | --- |
| General | Male | 563（27.1%） |
|  | Female | 1518（72.9%） |
| Ethnicity | Han ethnic group | 2002（96.2%） |
|  | Minority | 79（3.8%） |
| Birthplace | Urban | 759（36.5%） |
|  | Rural | 1322（63.5%） |
| Grade | Freshman | 1145（55.0%） |
|  | Sophomore | 587（28.2%） |
|  | Junior | 246（11.8%） |
|  | Senior | 103（4.9%） |
| Whether interact with autistic people | Yes | 320（15.4%） |
|  | No | 1761（84.6%） |
| Whether major in special education | Yes | 209（10.0%） |
|  | No | 1872（90.0%） |
| Whether take inclusive education courses | Yes | 148（7.1%） |
|  | No | 1933（92.9%） |
| type of major | Normal education students | 707（34.0%） |
|  | Non Normal education Students | 1374（66.0%） |

*Note:In the current cultural context of China, there are only two officially recognized genders. Therefore, gender is only set as male or female here;Ethnic groups are generally divided into Han and minority ethnic groups.*

**Table2. Model summary for regression predicting autism knowledge scores (*N* = 2,081)**

| **Model** | ***R*** | ***R*^2^** | **Adjusted *R*^2^** | **Standard Error of the Estimate** | **Durbin-Watson** |
| --- | --- | --- | --- | --- | --- |
| 1 | 0.431 | 0.186 | 0.183 | 3.87 | 1.86 |

*Note. Dependent variable: total knowledge score. R² indicates that 18.6% of the variance is explained by the model.*

*Durbin-Watson statistic indicates no autocorrelation in residuals.*

**Table 3. Model summary for regression predicting autism stigma scores (*N* = 2,081)**

| **Model** | ***R*** | ***R*^2^** | **Adjusted *R*^2^** | **Standard Error of the Estimate** | **Durbin-Watson** |
| --- | --- | --- | --- | --- | --- |
| 1 | 0.273 | 0.075 | 0.071 | 1.50 | 1.95 |

*Note. Dependent variable: total stigma score. R² indicates that 7.5% of the variance is explained by the model.*

*Durbin–Watson statistic indicates no serious autocorrelation in residuals.*

**Table 4. Regression coefficients and multicollinearity statistics for predictors of autism knowledge scores (*N* = 2,081)**

| coefficient a | | | | | | | |
| --- | --- | --- | --- | --- | --- | --- | --- |
| Model | Factors not normalized | | Normalization factor | *t* | *p* | Colinearity statistics | |
|  | *B* | *SE* | *β* |  |  | *Tolerance* | *VIF* |
| (constant) | 40.705 | 1.231 | — | 33.076 | 0.000 | — | — |
| Gender | 2.050 | 0.198 | 0.213 | 10.360 | 0.000 | 0.930 | 1.076 |
| Ethnic group | -0.373 | 0.445 | -0.017 | -0.836 | 0.403 | 0.991 | 1.009 |
| Place of birth | -0.192 | 0.177 | -0.022 | -1.082 | 0.279 | 0.985 | 1.015 |
| Grade | 0.401 | 0.118 | 0.082 | 3.411 | 0.001 | 0.687 | 1.455 |
| Whether interact with autistic people | -0.445 | 0.247 | -0.038 | -1.801 | 0.072 | 0.905 | 1.105 |
| Whether major in special education | -4.796 | 0.332 | -0.337 | -14.425 | 0.000 | 0.719 | 1.391 |
| Whether or not you have taken integrated education | -0.871 | 0.424 | -0.052 | -2.052 | 0.040 | 0.604 | 1.655 |
| Teacher professional or not | 0.401 | 0.224 | 0.044 | 1.791 | 0.073 | 0.638 | 1.567 |

*a. Dependent variable: Total score*

*Note. Dependent variable: total autism knowledge score. VIF = Variance Inflation Factor; SE = standard error; β = standardized coefficient. All VIF values < 5, indicating no multicollinearity. Significant results are bolded.*

**Table5. Demographic variables and comparison of means between groups(*N* = 2,081)**

| **Characteristics** |  | **Total knowledge score (D + E+T)**  ***M(SD)*** | **Stigma**  ***M(SD)*** |
| --- | --- | --- | --- |
| Gender |  | *t*=-11.633 | *t*=-5.979 |
|  |  | *p*<0.001 | *p*<0.001 |
|  |  | *d*=-0.574 | *d*=-0.295 |
|  | Male | 31.54（4.272） | 3.1030（1.546） |
|  | Female | 33.92（4.096） | 3.5586（1.544） |
| Ethnicity |  | *t*=1.600 | *t*=-1.076 |
|  |  | *p*=0.110 | *p*=0.282 |
|  |  | *d*=0.183 | *d*=-0.123 |
|  | Han ethnic group | 33.30（4.269） | 3.4281（1.563） |
|  | Minority | 32.52（4.414） | 3.6203（1.408） |
| Birthplace |  | *t*=0.250 | *t*=0.572 |
|  |  | *p*=0.803 | *p*=0.567 |
|  |  | *d*=0.011 | *d*=0.026 |
|  | Urban | 33.30（4.284） | 3.4611（1.589） |
|  | Rural | 33.26（4.273） | 3.4206（1.539） |
| Grade |  | *F*（3,2077）=7.410 | *F*（3,376.176）=3.293 |
|  |  | *p*<0.001 | *p*=0.021 |
|  |  | *η*²=0.011 | *η*²=0.005 |
|  | Freshman | 32.93（4.069） | 3.3520（1.508） |
|  | Sophomore | 33.47（4.490） | 3.5315（1.568） |
|  | Junior | 33.87（4.471） | 3.4593（1.711） |
|  | Senior | 34.50（4.392） | 3.7573（1.599） |
| Whether interact with autistic people |  | *t*=4.989 | *t*=3.887 |
|  |  | *p*<0.001 | *p*<0.001 |
|  |  | *d*=0.343 | *d*=0.256 |
|  | Yes | 34.51（4.921） | 3.7719（1.711） |
|  | No | 33.05（4.110） | 3.3742（1.520） |
| Whether major in special education |  | *t*=20.813 | *t*=12.870 |
|  |  | *p*<0.001 | *p*<0.001 |
|  |  | *d*=1.287 | *d*=0.827 |
|  | Yes | 37.89(3.297) | 4.5598(1.307) |
|  | No | 32.76(4.058) | 3.3098(1.532) |
| Whether take inclusive education courses |  | *t*=5.421 | *t*=2.344 |
|  |  | *p*<0.001 | *p*=0.020 |
|  |  | *d*=0.505 | *d*=0.232 |
|  | Yes | 35.26（4.665） | 3.7703（1.822） |
|  | No | 33.12（4.207） | 3.4097（1.533） |
| type of major |  | *t*=9.202 | *t*=5.801 |
|  |  | *p*<0.001 | *p*<0.001 |
|  |  | *d*=0.437 | *d*=0.275 |
|  | Normal education students | 34.48（4.403） | 3.7157（1.620） |
|  | Non Normal education Students | 32.65（4.073） | 3.2911（1.504） |

*Demographic variables and between-group mean comparison D=diagnosis, E=etiology, T=treatment, *p<0.05,**p<0.01*

*(Note:In the Chinese context, the division regarding ethnicity is generally Han Chinese and ethnic minorities.The place of birth, i.e., the civil registry, is divided into urban and rural areas).*

**Table 6.Spearman’s rank-order correlation between grade level and total ASD knowledge score (N = 2,081)**

| **Variable** | **Grade level** |
| --- | --- |
| Total Knowledge Score | 0.097** |

**Note.** Spearman’s *ρ* = .097. ***p*** < 0.01 (two-tailed). A positive value indicates that higher academic grade levels are associated with higher autism knowledge scores.

**Fig1. Percentage of students’ ways of acquiring ASD knowledge resources**
