# Supplementary material for: Knowledge and stigma of autism spectrum disorders in Chinese university students in the context of inclusive education
Source: PLoS One. 2025 Jun 26;20(6):e0320033. doi: 10.1371/journal.pone.0320033 (PMC12200771; doi:10.1371/journal.pone.0320033)
Supplement: Attachment — Submitted filename: Response to Reviewers.doc [file pone.0320033.s003.doc]

**Note on the Revised Manuscript with Marked Changes**

The revised Word document includes detailed responses to the reviewers' comments, marked using color-coded text for clarity:

Blue font indicates responses to Reviewer 1.

Green font indicates responses to Reviewer 2.

Purple font indicates content addressing comments raised by both reviewers.

For ease of reference:

Comments are labeled according to the reviewer and the section. For example, “reviewer1-1” refers to the first comment from Reviewer 1, and “reviewer2-A-1” refers to the first comment from Reviewer 2 under Section A.

Red bold text highlights revisions that directly correspond to specific reviewer comments.

We hope this format helps facilitate a clear and efficient review process. Please let us know if any further formatting adjustments are needed.

**Reviewer #1:** This study explores the knowledge and stigma of autism spectrum disorders (ASD) among Chinese university students, an important and timely topic in the field of inclusive education. The research is based on a large dataset (N=2,081). However, several methodological, statistical, and presentation issues deemed the manuscript not to be considered for publication in PLOS ONE.

1. The manuscript is not statistically sound. ANOVA is used to compare groups, but it does not control for confounding variables like gender, major, or prior ASD interaction. Regression models should be applied to ensure robustness.

**Response:**Thank you for your valuable suggestion regarding statistical rigor. While the primary analyses in our study employed ANOVA to assess group differences, we agree that it is important to examine the independence and potential collinearity among predictor variables.

To address this, we conducted additional linear regression analyses with the total knowledge score and the total stigma score as dependent variables. The results showed acceptable levels of explanatory power (*R²* = 0.186 for knowledge and *R²* = 0.075 for stigma), indicating that the selected predictors collectively account for a meaningful proportion of variance.

Importantly, we also tested for multicollinearity using the Variance Inflation Factor (VIF), and all VIF values were below the conventional threshold of 5 (ranging from 1.009 to 1.655). The Durbin-Watson statistics were 1.855 (knowledge) and 1.945 (stigma), suggesting no serious autocorrelation in the residuals. These results demonstrate that the independent variables are not collinear and can be interpreted as statistically independent in the context of the ANOVA tests.

We have added a summary of this robustness check in the revised Results section (**page 10,lines155-174)** and clarified this methodological decision in the Discussion **(page 13,lines226-234)** as a limitation and direction for future research. Thank you again for helping us strengthen the analytic transparency of our work.

2. The authors did not mention any selection criteria and sampling method. Again the manuscript states that data was collected via WeChat/QQ social groups, but this method may exclude students with limited internet access or those less interested in ASD topics, leading to self-selection bias.

**Response:**Thank you for your thoughtful comment. We have added a detailed description of the sampling method and participant recruitment procedures in the revised Participants and Procedures subsections of the Methods section. **(page7 lines92-107)**

Participants were recruited through university-affiliated WeChat and QQ groups, which are commonly used communication platforms among Chinese university students for course coordination, announcements, and academic surveys. Given their widespread use across higher education institutions in China, limited internet access is unlikely to have been a significant barrier to participation.

However, we acknowledge the possibility of self-selection bias, as students who were less interested in autism-related topics may have been less likely to engage with the survey. This potential limitation has been explicitly addressed in the revised Discussion section (Page14,Lines302-309), where we discuss its implications for generalizability and suggest that future studies incorporate sampling strategies that proactively engage a broader range of student interests.

3. The manuscript claims to use a Chinese version of the ASK-Q, but no details are provided on validation. How was it translated and tested for reliability? If it was previously validated, a citation should be provided.

**Response:** Thank you for pointing this out. The Chinese version of the ASK-Q survey was meticulously translated in accordance with a rigorous translation-retranslation protocol. The meticulously adapted Chinese ASK-Q has demonstrated robust psychometric properties across a substantial sample of Chinese respondents(Yu et al., 2020).This information has been added to the “Materials” subsection of the Methods section. **(page 9,lines128-137)**

4. The introduction discusses stigma but does not define how it was conceptualized and measured. Was stigma measured attitudinally, behaviorally, or structurally?

**Response:** We appreciate this observation. In the revised Introduction, we have added a definition of stigma based on the ASK-Q framework, which primarily captures attitudinal dimensions of stigma (e.g., perceptions and beliefs about autism).

Among the 48 items on the ASK-Q, 7 knowledge items also assess a sub-construct of ASD stigma (e.g., “ASD is caused by cold, rejecting parents”). The ASK-Q uses a binary system with options of correct and incorrect.On the stigma total score, a higher score represents a lower level of stigma endorsement. This clarification is now included on **pages4,lines38-45.**

5. The manuscript claims a "positive correlation" between grade level and ASD knowledge scores (*p*<0.001). However, ANOVA does not measure correlation—a Pearson or Spearman correlation test should be conducted instead.

**Revised Response:** Thank you for identifying this important distinction in statistical interpretation. We acknowledge that ANOVA is not an appropriate method for assessing correlation between two variables. In response to your suggestion, we conducted a Spearman’s rank-order correlation analysis to evaluate the relationship between academic grade level (treated as an ordinal variable) and total ASD knowledge scores.

The analysis revealed a small but statistically significant positive correlation (*ρ* = 0.097, *p* < 0.01, two-tailed), indicating that higher academic grades are modestly associated with greater knowledge about autism. This result is now appropriately reported in the revised Results section **(page11,lines193-197)**, and the previous misuse of the term “positive correlation” in the context of ANOVA has been corrected.

Thank you again for helping us improve the statistical clarity and accuracy of our manuscript.

6. The manuscript only reports p-values but does not provide effect sizes (Cohen’s *d*, *η²*, or *R²*). Effect sizes should be reported to show practical significance.

**Revised Response:** Thank you for raising this important point regarding the need to report effect sizes in addition to statistical significance. We have revised the **Results section** to include Cohen’s d for all independent-samples t-tests and partial eta squared (*η²*) for one-way ANOVA analyses, as appropriate.

These effect sizes are now reported alongside p-values in both the text and the corresponding tables. For example: The difference in total knowledge scores between male and female students yielded a medium effect size (*d* = 0.574), while the difference in stigma scores was smaller (*d* = 0.295). The impact of taking inclusive education courses showed a moderate effect on knowledge scores (*d* = 0.505) and a smaller but significant effect on stigma scores (*d* = 0.232). Major differences were most pronounced among students majoring in special education, with large effect sizes for both knowledge (*d* = 1.287) and stigma (*d* = 0.827). ANOVA results revealed small effect sizes across grade levels for knowledge (*η²* = 0.011) and stigma (*η*² = 0.005), indicating a modest impact of academic level on these outcomes.

These additions provide a clearer understanding of the practical significance of our findings and improve the transparency and interpretability of the statistical results. Revisions have been included throughout the results section**(pages11-12)** and are highlighted in **Table 5**. Thank you again for this valuable recommendation.

Reviewer #2: Thank you for the opportunity to review this manuscript, which investigates knowledge and stigma surrounding autism among Chinese student populations. I found the paper to be interesting and important, and I believe it has strong potential for publication following minor revisions. One of the strengths of the paper is its succinctness; I therefore offer my suggestions with the intention of supporting clarity, conceptual precision, and consistency—both in terms of scientific framing and language.

I have divided my comments into two broad sections: (1) Scientific and Conceptual Feedback and (2) Spelling and Grammar.

Please note that I have not been able to locate the dataset associated with this manuscript. As such, I have responded “no” to the data availability question in the review form. If the dataset is available for review or will be made available upon publication, this should be clearly stated in the manuscript and/or accompanying materials.

**1. Scientific and Conceptual Feedback**

**A. Overall Framing and Language**

1.The introduction could benefit from a more structured and purposeful opening. I suggest beginning with a clear statement of the broader issue—namely, the increasing number of autistic children requiring educational support—and then narrowing in on the implications for teacher preparedness and societal knowledge about autism. This would help establish a strong rationale for why autism knowledge and stigma in student populations is a crucial area of study, particularly in light of government initiatives to promote inclusive education.

2. The manuscript refers to autism as a “mental health issue.” While there is ongoing debate around the best way to frame autism, many autistic people and advocates regard it as a naturally occurring neurotype rather than a mental health condition. Framing autism as a disorder or pathology can contribute to stigma, particularly in the context of a paper explicitly addressing stigmatizing attitudes. Given the topic of the study, it is important to critically reflect on the terminology used, including the continued reference to “Autism Spectrum Disorder,” and consider aligning with neurodiversity-affirming language where appropriate.

3.There’s a sentence about the CDC prevalence data—please clarify what country this data refers to, especially since the study is based in China. It might also be worth including a sentence or two earlier on to set the Chinese educational context around autism (e.g., whether inclusive education is standardised, common, or still developing).

**ResponseA:** Thank you very much for your insightful feedback on the introduction. In response to your suggestions, we have undertaken a substantial revision of this section to enhance its structure, clarity, and conceptual framing.

1.Opening structure: We have reorganized the introduction to begin with a broader global framing of the increasing prevalence of autism and its implications for education systems. From there, the focus narrows to the challenges faced by general education teachers, the importance of pre-service preparation, and the relevance of student knowledge and attitudes. This revised structure provides a clearer rationale for investigating autism knowledge and stigma among university students. **（page3-5）**

2.Terminology and framing: We appreciate your point about the importance of language. We have removed the reference to autism as a “mental health issue” and now describe it as a “neurodevelopmental condition” or, where appropriate, as a “naturally occurring neurotype.” We also acknowledge ongoing debates around terminology and the implications of framing, particularly in the context of stigma, and have added a brief reflection on this issue in the revised introduction.**(page 3, line20)**

3.Clarification of CDC data: We have revised the sentence referencing CDC prevalence data to clearly indicate that the data refer to the United States. **(page 3, line21)**Additionally, we have incorporated recent prevalence estimates for China to provide a more balanced and locally relevant context.**(page3-4, lines22-24)**

4.Chinese educational context: A new sentence has been added to explain that inclusive education in China has been formally promoted through national policy, but its implementation remains uneven**(page4,line27-30)**. This provides international readers with a clearer understanding of the educational backdrop against which the current study is situated.

These revisions are reflected in the updated Introduction section (pages3-5). Thank you again for your constructive recommendations, which have helped us improve both the precision and accessibility of the manuscript.

**B. Clarity in Methods and Measures**

1. The classification of participant groups, beginning around line 52, requires significant clarification. The terms “normal-education students” and “non-normal-education students” are unclear and potentially problematic. It is essential to specify whether these refer to:

• Student teachers preparing to teach in general versus special education settings;

• Students with versus without special educational needs;

• Or another classification altogether.

**Response:** Thank you for this important observation. We agree that the terms “normal-education students” and “non-normal-education students” require clarification for an international readership. In the revised Participants subsection of the Methods section (Page 6,Lines80-90), we have now clearly defined these categories in the context of Chinese higher education. Specifically:

Normal-education students refer to students enrolled in teacher education programs at “normal universities” in China. These students are pre-service teachers preparing for careers in general education settings and receive formal training in pedagogy and, in some cases, inclusive education.

Non-normal-education students refer to students from academic disciplines outside the field of education (e.g., business, law, engineering). These students do not receive teacher training and are not preparing for educational careers.

We have retained these original terms to reflect common usage in Chinese educational discourse but have now added explanatory language to ensure clarity and avoid misinterpretation. This distinction supports the purpose of our study, which is to compare autism knowledge and stigma between students with and without professional educational training backgrounds.

Thank you again for prompting us to clarify this important classification.

**2.Furthermore,** the term “subjects” should be replaced with “participants,” not only because it is a more respectful and contemporary term, but also to avoid confusion with “subjects” in the academic sense (e.g., disciplines or courses of study).

**Response:** We have replaced all instances of the term “subjects” with “participants” to reflect current academic standards and promote respectful language.

**3.It wasn’t clear** to me whether participants were asked if they themselves are autistic—if not, it might be worth noting this as a limitation.

**Response:** Thank you for raising this point. We confirm that participants were not asked whether they themselves are autistic. In the context of mainland China, autism diagnoses remain primarily focused on children, and formal diagnostic pathways for adults are limited. Due to institutional barriers such as the high-stakes college entrance examination system and a lack of inclusive policies in higher education, autistic university students are extremely rare and not widely reported. Nevertheless, we acknowledge this as a limitation and have added a note in the Discussion section (**pages13-14,lines238-243)**, stating that the possible inclusion of undiagnosed autistic participants cannot be ruled out and may merit further exploration in future studies.

**4.The phrase “QQ social group”** on line 83 might not be familiar to international readers—could you clarify what this means? (e.g., a social media platform?)

**Response:** Thank you for your helpful suggestion. We have revised the Methods section to clarify that “QQ social groups” refer to group chats established on **Tencent QQ**, a widely used instant messaging and social networking platform in China. QQ is commonly used by university students to form class groups and academic communities for communication and coordination. In this study, survey invitations were distributed through these QQ groups, which serve as the primary mode of digital interaction among Chinese students in higher education. This explanation is now included in the Procedures subsection **(page 7 lines 96-101)** to ensure accessibility for international readers.

**5.The ASK-Q scale should be cited on first mention.**

**Response:** We have added a full citation for the ASK-Q upon first mention in the Materials subsection, citing both Harrison et al. (2017) and Harrison et al. (2019), in accordance with your recommendation.

**These revisions are on page8,lines110-114**

**6.It would also be helpful to know whether participants were compensated in any way.**

**Response:** We have clarified in the Procedures subsection that participants received no monetary or material compensation for their participation. Participation was entirely voluntary and anonymous. These revisions are on **page7,lines103-104**

**7.The methods section is currently a bit dense—perhaps consider splitting it into subsections like Participants, Materials, Procedure, and Data Analysis to make it easier to follow.**

**Response:** To improve clarity and readability, we have revised the Methods section and reorganized it into four standard subsections: Participants, Materials, Procedure, and Data Analysis. This new structure improves the logical flow of information and aligns with conventional academic reporting formats.These changes are now reflected throughout the revised Methods section **(pages6-9)**. Thank you again for helping us improve the methodological transparency and readability of our manuscript.

**C. Results, Analysis, and Interpretation**

**1.Some of the p-values were hard to interpret (e.g., lines 112–114)—I wasn’t always sure what the test was comparing (e.g., is the p-value comparing stigma levels between groups, or knowledge levels?). It might be helpful to make these tests clearer in the text.**

**Response:** We have revised the Results section to ensure that each reported *p*-value is explicitly tied to a clearly stated comparison—whether between groups for ASD knowledge or stigma, and whether based on gender, academic background, contact with autistic individuals, or training variables. Each test result now specifies the dependent variable (knowledge or stigma), the statistical method (t-test, ANOVA, or correlation), and the associated effect size (e.g., Cohen’s *d*, partial *η²*), to improve clarity and interpretability.  **These revisions are on Pages11-13.**

**2.In terms of formatting, be consistent with the number of decimal places, and spell out all statistics.**

**Response:** All statistical results now follow a consistent format. Means and standard deviations are reported to two decimal places, p-values are reported to three decimal places (e.g., *p* = 0.001), and all statistical terms (e.g., *t, F, ρ, η², d*) are fully spelled out and italicized, following APA and journal guidelines.

**3.The number of participants who failed attention checks seems relatively high in proportion to the total sample. This section would benefit from context—e.g., how many checks were included, what they involved, and whether there were any identifiable patterns in the failures (e.g., demographic skew). This would help reassure readers of the robustness of the remaining dataset.**

**Response:**To ensure data quality and minimize random or inattentive responses, we embedded three attention-check items within the questionnaire. These included instructions such as “Please select option 2 for this question”, which served as a simple measure to assess whether participants were reading the items carefully. Responses that failed any of these designated checks or were incomplete were excluded from the final dataset. In total, 483 responses were removed based on these criteria, resulting in 2,081 valid responses with an effective response rate of 81.2%. To further address concerns about potential sampling bias, we examined the demographic characteristics of the excluded cases and found no discernible patterns of skew in terms of gender, academic background, or grade level.

We have added this explanation to the Participants subsection of the Methods section to clarify the rationale and implementation of our data screening process and to reinforce the robustness of the retained dataset. **These revisions are on page 6, lines 74-79.**

**4.In the results section, some of the analyses and findings could be linked back more clearly to the hypotheses or research questions.**

**Response:**

We have revised the final sentences of each major results subsection to explicitly link the findings to our hypotheses. For example, comparisons between special education majors and non-education majors directly support our hypothesis that professional training in education—especially in special or inclusive education—is associated with higher ASD knowledge and lower stigma. **These links are now highlighted throughout the Results and elaborated upon in the Discussion section.**

Additional robustness checks and confounding variable control: In response to suggestions regarding confounding variables, we have conducted supplementary multiple linear regression analyses using total knowledge and total stigma scores as dependent variables, with demographic and academic factors as predictors. The results (*R*² = 0.186 for knowledge, *R*² = 0.075 for stigma) support the independence of key predictors and the robustness of the primary group comparisons. We also included VIF values and Durbin-Watson statistics to rule out multicollinearity and autocorrelation.

These analyses and results are reported in the Additional Analyses subsection of the Results section and presented in **Tables1-3**. We believe these revisions significantly improve the clarity, transparency, and theoretical alignment of the Results section. Thank you again for your detailed and constructive comments.

**D. Discussion and Implications**

The discussion section has some nice points, especially around the importance of exposure and experience in reducing stigma. However, I’d be a bit cautious with how some findings are phrased.

It might also be worth commenting on how autism knowledge is often contested or variable—some sources of “knowledge” (e.g., clinical versus lived experience accounts) may convey very different messages. You could briefly acknowledge this and suggest future research could look more closely at what kind of knowledge reduces stigma most effectively.

**Response:**

Thank you for this thoughtful and nuanced suggestion. We agree that autism knowledge is not a monolithic construct and that different types of knowledge—such as clinical, educational, or lived experience-based perspectives—may carry distinct implications for how autism is understood and how stigma is shaped.

In response, we have revised the Discussion section to acknowledge this conceptual complexity. Specifically, we now note that while increasing overall knowledge is generally associated with reduced stigma, the content and source of that knowledge may matter significantly. For example, clinical descriptions may unintentionally emphasize deficits, whereas narratives grounded in lived experience or neurodiversity frameworks may foster more inclusive and empathetic perspectives.

We also highlight this as a potential direction for future research: to investigate which types of autism-related knowledge—formal (e.g., medical, academic) or informal (e.g., peer narratives, personal interaction)—are most effective in reducing attitudinal stigma among different learner populations.

This reflection has been incorporated into the revised Discussion section **(page 15-17).** Thank you again for encouraging us to broaden the conceptual depth of our analysis.

2. Spelling and Grammar

While the manuscript is generally clearly written, there are a few minor spelling and formatting issues that could be addressed:

• Line 24: “education’s” – missing the n.

• Lines 100–101: Missing space between words.

• Line 113: “the” may be unnecessary or redundant in this context.

• Line 204: “Reference” should be corrected to “References.”

• Table 1: “female” missing an e.

• Table Headings: Ensure consistent capitalisation across all tables.

**Response**: Thank you for pointing out these issues. Since the manuscript has been substantially revised for clarity and structure, all previously noted spelling, formatting, and typographical errors have been carefully reviewed and corrected throughout the revised version. We have also standardized table headings and ensured consistency in capitalization and terminology across all sections.

We appreciate your attention to detail, which helped us further improve the overall readability and presentation of the manuscript.
